# An Exploratory Study on Beneficial Effect of BE-FD-1 (Mineral-Enriched *Raphanus sativus* L. Leaf Extract) in High-Fat-Diet- and Streptozotocin-Induced Diabetic Mice

**DOI:** 10.3390/nu18111832

**Published:** 2026-06-05

**Authors:** Sung Jin Kim, Kyeong-No Yoon, Daewon Hwang, Jung Eun Park, Gabsik Yang, You Jeong Moon, Hyun Won Kim, Jeong Eun Jang, Ki Hyun Kim, Minjung Park, Ki Sung Kang

**Affiliations:** 1 Department of Korean Medicine, Gachon University, 1342 Seongnam-daero, Sujeong-gu, Seongnam-si 13120, Republic of Korea; sungjinkim001@gmail.com (S.J.K.); bgbg2020@gachon.ac.kr (D.H.); ppp1416@gachon.ac.kr (J.E.P.); yanggs@gachon.ac.kr (G.Y.); 2 Department of Dermatology, Seoul National University College of Medicine, Seoul 03080, Republic of Korea; kyeongnoyoon@snu.ac.kr; 3 BORNECO SYSTEMS Co., Ltd., 327 Jungang ro, Anseong-si 17579, Republic of Korea; borneco@bornecosystems.com (Y.J.M.); kimhw@hanmail.net (H.W.K.); 4 School of Pharmacy, Sungkyunkwan University, Suwon 16419, Republic of Korea

**Keywords:** diabetes mellitus, *Raphanus sativus*, high-fat diet, antidiabetic, insulin secretion

## Abstract

Background/Objectives: Type 2 diabetes mellitus (T2DM) is a chronic metabolic disorder associated with insulin resistance, β-cell dysfunction, and systemic complications. Methods: In this preliminary study, the metabolic effects of BE-FD-1, a water extract of *Raphanus sativus* L. leaves cultivated under a mineral-fortification protocol, were investigated in a high-fat-diet/streptozotocin (HFD/STZ)-induced diabetic mouse model. Inductively coupled plasma mass spectrometry analysis confirmed the presence of vanadium, chromium, magnesium, zinc, and calcium in radish leaf. Male C57BL/6 mice (n = 5/group) were orally administered BE-FD-1 at 250 or 500 mg/kg once daily for four weeks, with metformin (250 mg/kg) as a positive reference. Results: BE-FD-1 at 500 mg/kg significantly reduced the oral glucose tolerance test area under the curve and fasting blood glucose levels, significantly restored serum insulin levels, and significantly decreased serum ALT, triglyceride, and total cholesterol levels relative to the HFD/STZ control group. Body weight gain and AST showed non-significant decreasing tendencies. Serum creatinine remained within the normal range, providing a preliminary safety signal that should be interpreted with caution given the absence of additional renal biomarkers and histopathological evaluation. Conclusions: These exploratory findings suggest that BE-FD-1 may warrant further investigation as a candidate functional ingredient for T2DM-related metabolic dysfunction; however, larger studies with comprehensive phytochemical characterization, mechanistic validation, and broader safety evaluation are required.

## 1. Introduction

Type 2 diabetes mellitus (T2DM) is a chronic metabolic disorder characterized by progressive insulin resistance and pancreatic β-cell dysfunction, and sustained hyperglycemia leads to a wide range of life-threatening complications, including cardiovascular diseases, renal failure, neuropathy, and retinopathy [1,2]. According to the 11th edition of the International Diabetes Federation Diabetes Atlas (2025), approximately 589 million adults worldwide (one in nine) have diabetes, and this figure is projected to reach 853 million by 2050 [3,4]. Considering that more than 95% of all diabetes cases are classified as T2DM, the rapidly growing global socioeconomic burden of this disease underscores the urgent need for effective preventive and therapeutic strategies.

The pathophysiology of T2DM involves a complex interplay among chronic low-grade inflammation, oxidative stress, impaired insulin signaling, and ectopic lipid accumulation [5,6]. The animal model combining a high-fat diet (HFD) with low-dose streptozotocin (STZ) administration is one of the most widely employed preclinical models for T2DM, as it simultaneously recapitulates peripheral insulin resistance and pancreatic β-cell damage. HFD induces chronic adipose tissue inflammation and systemic insulin resistance, while STZ enters pancreatic β-cells via the GLUT2 transporter and selectively alkylates their DNA, thereby impairing insulin secretory capacity; the combination of these two insults stably reproduces a metabolic state closely resembling clinical T2DM [7,8].

Metformin, a biguanide compound, is the globally recommended first-line pharmacological treatment for T2DM and primarily acts by suppressing hepatic gluconeogenesis and activating the AMP-activated protein kinase (AMPK) pathway to improve peripheral glucose utilization. However, long-term metformin use is associated with adverse gastrointestinal effects, such as nausea, diarrhea, abdominal discomfort; vitamin B12 deficiency; and lactic acidosis in rare cases [9,10]. These limitations have fueled growing research interest in naturally derived functional materials that offer high safety profiles and can serve as complementary or alternative therapeutic agents [11,12].

Mucheong is a traditional Korean food prepared by naturally sun-drying the leaves and stalks of green radish (*Raphanus sativus* L.). During the drying process, dietary fiber content increases more than 3- to 4-fold compared to the fresh state, and bioactive compounds, including polyphenols, glucosinolates, β-carotene, chlorophyll, calcium, and iron, become highly concentrated [13,14]. The leaves of the radish plant are considered nutritionally superior to the roots, and the bioactive compounds present in Mucheong have been reported to exert antidiabetic effects through multiple mechanisms. Radish leaf extracts have been shown to inhibit α-glucosidase activity, thereby reducing postprandial glucose absorption, and to suppress the formation of advanced glycation end-products (AGEs), which are key mediators of diabetic complications [15,16]. Glucosinolate hydrolysis products, particularly isothiocyanates, have been demonstrated to exert anti-inflammatory effects and improve insulin sensitivity [17], whereas the abundant dietary fiber in Mucheong contributes to delayed gastric emptying and reduced glycemic index, thereby supporting glycemic control [18]. Furthermore, Mucheong extracts have been reported to reduce serum triglyceride and total cholesterol levels and promote adiponectin synthesis, thereby improving lipid metabolism [19,20].

Recently, vanadium has attracted considerable attention as a trace mineral with potential benefits for diabetes management. Vanadium exhibits insulin-mimetic properties and may contribute to lowering blood glucose levels by activating insulin signaling pathways and facilitating cellular glucose uptake [21,22]. Consequently, it is highly efficacious in cases of insulin resistance. Furthermore, it has drawn considerable interest from the medical community as a critical substance for patients with diabetes, which is characterized by the inability of pancreatic beta cells to synthesize insulin or their resistance. However, excessive vanadium intake can induce adverse effects, including gastrointestinal disturbances, hepatotoxicity, and neurological complications. Moreover, its clinical application is hindered by the inefficiencies arising from its low systemic absorption rate and poor bioavailability [23].

Vanadium has attracted considerable attention as a trace mineral with insulin-mimetic properties, and chromium, zinc, calcium, and magnesium have also been implicated in glucose and metabolic regulation [24,25]. However, the direct use of such minerals may be limited by concerns regarding bioavailability, tolerability, and dose-dependent toxicity. In this context, crop-based mineral fortification may provide an alternative strategy for delivering metabolically relevant minerals in a food-derived matrix.

BE-FD-1 is a water extract derived from dried leaves of *Raphanus sativus* L. cultivated using a fortified mineral mixture composition. Based on the known metabolic activities of radish-derived phytochemicals and diabetes-related minerals, we hypothesized that BE-FD-1 would improve glucose homeostasis and related metabolic abnormalities in HFD/STZ-induced diabetic mice. Therefore, the aim of the present study was to evaluate the antidiabetic efficacy of BE-FD-1 in a mouse model of HFD/STZ-induced type 2 diabetes by assessing body weight change, fasting blood glucose, glucose tolerance, serum insulin, and selected biochemical parameters.

## 2. Materials and Methods

### 2.1. Plant Material and Cultivation Conditions

Green radish (*R. sativus* L.) plants treated with the BE-FD-1 mineral mixture were cultivated until 2024 in an open field at the foot of Mount Cheongtae (Dunnae-myeon, Hoengseong-gun, Republic of Korea). The cultivation site, managed by the Cheongtaesan Agricultural Corporation, encompasses a total area of 1.5 hectares. Throughout the experimental period, all basic agricultural practices, including soil management, irrigation, and weed and pest control, strictly complied with established organic farming standards.

### 2.2. Composition and Application Strategy of the BE-FD-1 Mineral Mixture

The BE-FD-1 mineral mixture was formulated primarily with vanadium (V) and trivalent chromium (Cr^3+^) ions, supplemented with zinc (Zn), calcium (Ca), and magnesium (Mg) ions. Based on the established patent specifications for this composition, the mixture was formulated with specific weight ratios: V:Cr = 1:0.05–0.2, V:Cr:Zn = 1:0.05–0.2:0.5–1.5, and Ca:Mg = 1:0.2–0.8. The recommended concentration of BE-FD-1 ranges from 10 to 30 ppm. Concentrations exceeding this optimal range (e.g., ≥50 ppm) have been noted to potentially induce phytotoxicity, characterized by growth retardation, decreased chlorophyll content, and chlorosis. Consequently, the standard operating procedure for this open-field study was designed for repeated applications at low concentrations. Furthermore, the application dosage was strategically reduced during the latter half of the cultivation period, guided by intermediate quality control (QC) mineral analysis results, to optimize plant uptake and prevent toxicity.

In this study, V was used as a key management indicator, and the results of the first inductively coupled plasma mass spectrometry (ICP-MS) were fed back into the cultivation operations. As the V content was confirmed in the first analysis in early October, the frequency of foliar application was reduced by more than half starting around mid-October, and adjustments were made to almost completely stop foliar application during the latter half of the cultivation (component-based feedback control).

### 2.3. Preparation and Handling of BE-FD-1 and Its Subsequent Cultivation (BE-FD-sc)

BE-FD-1 was prepared as a water extract derived from dried leaves of *Raphanus sativus* L. cultivated under the BE-FD-1 mineral-fortification protocol. The dried leaves of the plant (122 g) were subjected to hot water extraction with 700 mL of distilled water twice at 60 °C for 3 h each. After extraction, the mixture was filtered, concentrated under reduced pressure, and lyophilized (freeze-dried) to yield 4.9 g of the final extract powder (extraction yield: 4.0%, *w*/*w*). The prepared BE-FD-1 material was stored under refrigerated conditions and dissolved in sterile distilled water immediately before oral administration. Detailed extraction parameters, including extraction temperature, extraction time, extraction yield, and long-term stability, were not fully recorded or evaluated in the present preliminary study.

A subsequent cultivation experiment was conducted in the following year after completion of the mineral-enrichment process used for BE-FD-1 production. The primary objective of this study was to evaluate the influence of residual minerals remaining in the soil after termination of the BE-FD-1 manufacturing cultivation system. Following the final harvest of BE-FD-1, the mineral-enrichment treatment program was discontinued, and no additional mineral supplementation was applied during the next cultivation cycle. The same agricultural soil was reused in order to investigate whether residual minerals accumulated during the previous BE-FD-1 cultivation period could affect soil properties, plant growth, or mineral uptake in the subsequent year.

### 2.4. Determination of Mineral Content via ICP-MS

To determine the mineral composition of the radish leaf samples, ICP-MS was employed following a wet chemical digestion process. The quantitative analysis of specific macro- and trace elements, namely vanadium (V), chromium (Cr), magnesium (Mg), zinc (Zn), and calcium (Ca), was performed by the Korea Institute of Ceramic Engineering and Technology (KICET, Jinju-si, Republic of Korea). The elemental concentrations were measured using a PerkinElmer NexION^®^ 2000 ICP-MS system (PerkinElmer, Waltham, MA, USA).

The analysis was conducted in two distinct phases: an initial evaluation for internal quality control (QC-1) and a subsequent verification for external partner submission (QC-2). ICP-MS analysis was also conducted for non-fortified radish, BE-FD-1 and BE-FD-1-sc.

### 2.5. UPLC-Q-TOF MS Analysis

UPLC–Q–TOF MS analysis was performed using an Agilent G6545B quadrupole time-of-flight (Q–TOF) mass spectrometer (Agilent Technologies, Santa Clara, CA, USA) coupled to an Agilent 1260 Infinity II system. BE-FD-1 extract was weighed and dissolved in 100% methanol, then filtered through a 0.45 µm hydrophobic polytetrafluoroethylene (PTFE) membrane filter prior to analysis. A 3 µL aliquot of the BE-FD-1 extract (5000 ppm) was injected onto an ACQUITY UPLC BEH C18 Column (2.1 mm × 150 mm; 1.7 μm; Waters corp.) maintained at 30 °C. The mobile phase consisted of 0.1% (*v*/*v*) formic acid in water (A) and 0.1% (*v*/*v*) formic acid in methanol (B), delivered at a flow rate of 0.3 mL/min under the following gradient conditions: 5–60% B over 10 min, maintained isocratically at 100% B for 2 min, and re-equilibrated at 5% B for 3 min. The MS and MS/MS parameters were set as follows: positive ionization mode; gas temperature, 320 °C; drying gas (N_2_) flow rate, 8 L/min; nebulizer pressure, 35 psi; sheath gas temperature, 350 °C; sheath gas flow rate, 11 L/min; capillary voltage, 3500 V; nozzle voltage, 1000 V; fragmentor voltage, 150 V; MS range, *m*/*z* 100–1700; MS acquisition rate, 1 spectrum/s; acquisition time, 1000 ms/spectrum; MS/MS range, *m*/*z* 50–500; MS/MS acquisition rate, 1 spectrum/s; acquisition time, 1000 ms/spectrum; and fixed collision energy, 40 eV. Internal reference compounds (purine and HP-0921) were used for real-time mass calibration. The reference ions in the positive ion mode were observed at *m*/*z* 118.0862 and 922.0097.

### 2.6. Total Flavonoid Content (TFC)

The total flavonoid content was determined using the aluminum chloride colorimetric method. The samples were prepared in distilled water at concentrations of 10, 5, and 2.5 mg/mL. An aliquot of each sample solution was mixed with aluminum chloride reagent and allowed to react at room temperature. After incubation, the absorbance was measured using a spectrophotometer.

Quercetin was used as a standard to construct a calibration curve, and the total flavonoid content was calculated accordingly. The results were expressed as μg quercetin equivalents per mL of sample solution (μg QE/mL). All experiments were performed in triplicate. All data are presented as mean ± standard deviation (SD) of three independent experiments.

### 2.7. Total Polyphenol Content (TPC)

The total polyphenol content was determined using the Folin–Ciocalteu method. The samples were prepared in distilled water at concentrations of 10, 5, and 1 mg/mL. An aliquot of each sample solution was mixed with 2 N Folin–Ciocalteu reagent and allowed to react at room temperature, followed by the addition of 7.5% sodium carbonate solution. The mixture was then incubated in the dark for 1 h.

After incubation, the absorbance was measured at 765 nm using a spectrophotometer. Gallic acid (5–80 μg/mL) was used as a standard to construct a calibration curve, and the total polyphenol content was calculated accordingly. The results were expressed as mg gallic acid equivalents per gram of dry matter (mg GAE/g DM). All experiments were performed in triplicate. All data are presented as mean ± standard deviation (SD) of three independent experiments.

### 2.8. Animals and Experimental Conditions

Six-week-old male C57BL/6 mice (approximately 20 g) were purchased from Orient Bio, Inc. (Seongnam, Republic of Korea). The animals were allowed to acclimatize for one week prior to the experiment. All experiments were conducted in Laboratory Room 101 of the College of Korean Medicine, Gachon University. Mice were maintained under controlled conditions (23 ± 3 °C, 55–70% relative humidity, and a 12 h light/dark cycle; lights on from 09:00 to 21:00). Food and water were provided ad libitum. All the experimental procedures were approved by the Institutional Animal Care and Use Committee (IACUC) of Gachon University (approval no. GU1-2025-IA0038-00) and conducted in accordance with the institutional guidelines for animal care.

### 2.9. Induction of Diabetes by High-Fat Diet and Streptozotocin

**Figure 1:**
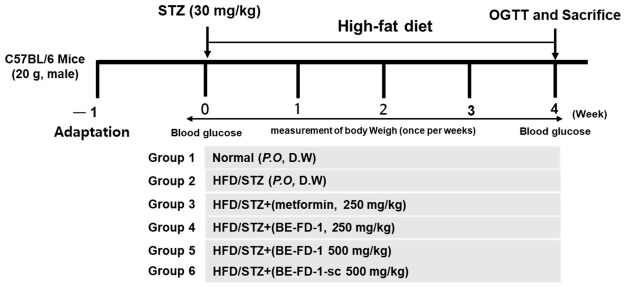
Schematic diagram of the experimental design and treatment schedule. BE-FD-1 was stored under refrigerated conditions and dissolved in sterile distilled water immediately before oral administration. The normal and HFD/STZ control groups received an equivalent volume of distilled water. Metformin was used as a positive control at 250 mg/kg. All treatments were administered orally once daily for 4 consecutive weeks. Black double-headed arrow: oral administration period of the test sample.

Diabetes was induced using a combination of a high-fat diet (HFD) and low-dose streptozotocin (STZ). After 1 week of acclimatization, mice in the normal control group were maintained on a normal diet (standard chow), whereas mice assigned to the diabetic groups were fed an HFD. STZ was freshly prepared in citrate buffer and administered intraperitoneally at 30 mg/kg twice at 1-week intervals. The HFD was maintained throughout the 4-week experimental and treatment period in the diabetic groups. The HFD was maintained throughout the diabetes induction and treatment periods. After the second STZ injection, mice were fasted for 16 h, and fasting blood glucose was measured using tail vein blood for confirmation of the diabetic phenotype and subsequent group allocation. The animals were then randomly assigned to the following groups (n = 5 per group): normal control, HFD/STZ diabetic control, metformin-treated group (250 mg/kg), BE-FD-1-treated group (250 mg/kg), BE-FD-1-treated group (500 mg/kg), and BE-FD-1-sc-treated group (500 mg/kg) (Figure 1). Doses were converted to human equivalent doses (HED) using the body surface area (BSA) normalization method [26,27].

### 2.10. Oral Glucose Tolerance Test

An oral glucose tolerance test (OGTT) was performed to evaluate the effect of BE-FD-1 on glucose tolerance. After the treatment period, the diabetic mice were fasted for 16 h and then orally administered a high-concentration glucose solution. Blood glucose levels were measured at 0, 30, 60, and 120 min using tail vein blood and a commercial glucose monitoring kit. A time–glucose curve was generated, and the area under the curve (AUC) was calculated. The results are presented in tabular and graphical form.

### 2.11. Measurement of Fasting Blood Glucose

To evaluate the long-term glycemic control effects of BE-FD-1 in HFD/STZ-induced diabetic mice, fasting blood glucose levels were measured after 16 h of fasting at the end of the 4-week treatment period using tail vein blood. After 16 h of fasting, blood samples were collected from the tail vein at designated time points, and blood glucose levels were determined. Changes in fasting blood glucose levels were analyzed and compared between groups.

### 2.12. Biochemical Analysis of Serum Parameters

At the end of the experimental period, blood samples were collected after fasting and centrifuged to obtain serum. Serum levels of AST, ALT, triglycerides, total cholesterol, and creatinine were analyzed by an external laboratory (GENIA, Seongnam, Republic of Korea). Serum insulin levels were measured using a mouse high-range insulin ELISA kit according to the manufacturer’s instructions. Serum insulin levels were measured using a mouse high-range insulin ELISA kit (ALPCO, Salem, NH, USA), according to the manufacturer’s instructions. Absorbance was measured at 450 nm, and the insulin concentration was calculated using a standard curve.

### 2.13. Statistical Analysis

Because the present study consisted of five independent experimental groups rather than a fully crossed factorial design, statistical comparisons for each endpoint were performed using one-way analysis of variance (ANOVA), followed by Tukey’s multiple comparison test. A *p*-value < 0.05 was considered statistically significant.

## 3. Results

### 3.1. Mineral Composition Profiles of Radish Leaf Samples

The mineral contents of radish leaf samples collected during the cultivation-stage QC process, as quantified by ICP-MS, are summarized in Table 1. All elemental concentrations are expressed in milligrams per kilogram (mg/kg). The quantitative results demonstrated some variation in the mineral profiles between the two phases. In the first quality control phase (QC-1; Report No. 2024-3345), the concentrations of V, Cr, Mg, Zn, and Ca were 14.1, 2.17, 2857, 88.6, and 5640 mg/kg, respectively. The second phase (QC-2; Report No. 2024-3684) revealed decreased levels of V (3.36 mg/kg), Cr (0.28 mg/kg), and Zn (32.1 mg/kg), accompanied by substantially higher accumulations of Mg (4304 mg/kg) and Ca (22,790 mg/kg). ICP-MS analysis confirmed the presence of vanadium, chromium, magnesium, zinc, and calcium in radish leaf samples collected during the cultivation process. The variability observed between the two QC phases may reflect differences in sampling time, cultivation stage, and agronomic conditions, all of which are known to influence mineral accumulation in biofortified crops [28]. These data were used primarily for cultivation-stage mineral verification rather than as a direct measure of in vivo mineral exposure following oral administration, because mineral concentration in plant tissue does not directly indicate bioaccessibility or bioavailability [29]. Nevertheless, detailed chemical characterization of the final BE-FD-1 extract was not performed in the present study, and major phytochemical constituents such as glucosinolates, polyphenols, and flavonoids were not quantitatively analyzed. Therefore, the reproducibility and pharmacological interpretation of BE-FD-1 should be considered with caution [30,31].

In addition to the two cultivation-stage QC analyses, comparative mineral profiling was performed for the non-fortified control sample, BE-FD-1, and BE-FD-1-sc (Table 2). BE-FD-1-sc was confirmed to be a subsequent-cultivation sample produced in the following year without additional mineral treatment after the initial BE-FD-1 mineral-fortification cultivation. Therefore, BE-FD-1-sc was interpreted as a subsequent-cultivation sample rather than a conventional non-fortified control sample.

Compared with the non-fortified control, BE-FD-1 showed higher levels of V, Cr, Mg, Zn, and Ca, supporting the successful alteration of the mineral profile by the BE-FD-1 mineral-fortification protocol. In contrast, BE-FD-1-sc showed markedly lower levels of V, Mg, and Zn than BE-FD-1, while Ca remained comparable and Cr was moderately reduced. The lower levels of several minerals in BE-FD-1-sc may be attributable to the absence of additional mineral treatment during the subsequent cultivation cycle. Together with the biological activity results of BE-FD-1-sc, these findings provide supportive information suggesting a possible association between cultivation-dependent mineral profile changes and biological activity.

These ICP-MS data were used for compositional verification of BE-FD-1-related samples, including the final administered BE-FD-1 batch; however, they do not directly demonstrate mineral bioaccessibility, bioavailability, or systemic mineral exposure after oral administration.

### 3.2. UPLC-QTOF-HRMS-Based Phytochemical Profiling, Total Flavonoid Content, and Total Polyphenol Content of BE-FD-1

**Figure 2:**
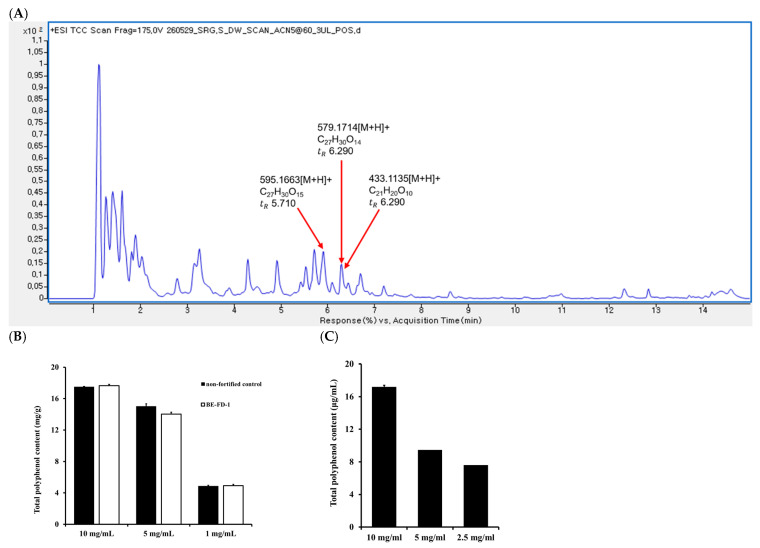
UPLC-QTOF-HRMS-based phytochemical profiling, total polyphenol and total flavonoid content of BE-FD-1. (**A**) Total ion chromatogram of BE-FD-1 obtained in positive electrospray ionization mode. (**B**) Total polyphenol content of BE-FD-1. (**C**) Total flavonoid content of BE-FD-1 determined using the aluminum chloride colorimetric method.

Qualitative analysis of the BE-FD-1 extract was performed using UPLC–Q–TOF–HRMS to obtain its chemical profile, revealing three putative flavonoids (Table 2). All detected compounds were compared with the experimental mass data, and the corresponding ppm error values were calculated. The compounds were then tentatively identified based on their mass spectral characteristics. A compound was considered a positive match when the difference between the theoretical and measured exact masses was within ±50 ppm. The exact masses were determined from the protonated molecular ions ([M + H]^+^) detected in positive electrospray ionization (ESI) mode (Figure 2A).

The total polyphenol contents of the non-fortified control and BE-FD-1 samples were compared, and the results showed little difference between the two groups (Figure 2B). These findings suggest that the mineral-enriched cultivation method had a minimal effect on the phenolic compound composition of radish leaves. Therefore, it was considered that mineral fortification cultivation did not significantly alter the total polyphenol content in radish leaves. Total flavonoid content (TFC) was measured using the aluminum chloride colorimetric method and expressed as μg quercetin equivalents per mL of sample solution (μg QE/mL). As shown in Figure 2C, the TFC of BE-FD-1 increased in a concentration-dependent manner. BE-FD-1 showed TFC values of 7.62 ± 1.71, 9.48 ± 0.31, and 17.20 ± 0.88 μg QE/mL at concentrations of 2.5, 5, and 10 mg/mL, respectively. Among the tested concentrations, the highest TFC value was observed at 10 mg/mL. These results indicate that BE-FD-1 contains flavonoid-related compounds, and that the measured flavonoid content increased according to sample concentration (Figure 2C).

### 3.3. Effect of BE-FD-1 on Body Weight

**Figure 3:**
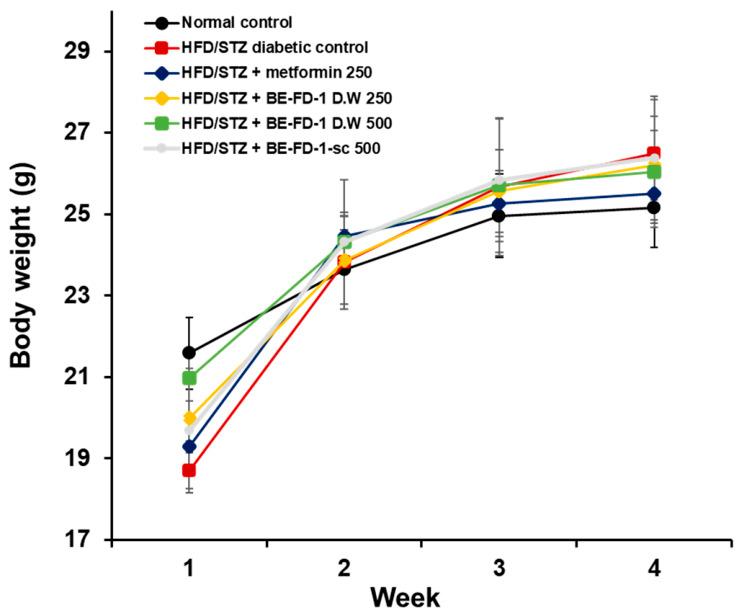
Effect of BE-FD-1 on body weight gain in STZ/HFD-induced diabetic mice. Body weight gain was measured during the experimental period in Normal, STZ/HFD, Metformin-treated, and BE-FD-1-treated groups (250 and 500 mg/kg). Data are presented as the mean ± SEM (n = 5 per group). Statistical significance was analyzed using one-way ANOVA followed by Tukey’s post hoc test. Some data points/lines overlap because the body weight values were similar among the groups; this does not affect the interpretation of the results.

During the experimental period, the STZ/HFD-induced diabetic group showed greater body weight gain than the normal control group. BE-FD-1 administration tended to attenuate body weight gain, and the BE-FD-1 (500 mg/kg) group showed a numerically lower body weight gain (5.1 g) than the STZ/HFD group (7.8 g). The metformin-treated group exhibited a body weight gain of 6.2 g. In contrast, BE-FD-1-sc did not markedly alter body weight gain compared with the STZ/HFD group. Where statistical significance was not observed, the differences were interpreted as tendencies only (Figure 3).

### 3.4. Effect of BE-FD-1 on Oral Glucose Tolerance Test

**Figure 4:**
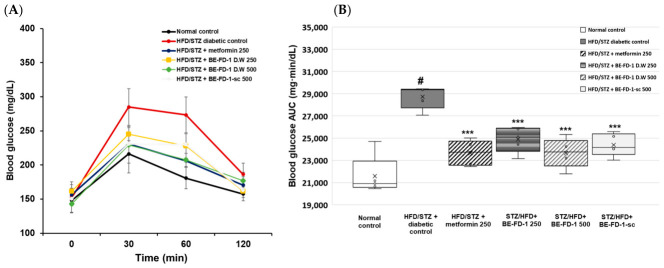
Effect of BE-FD-1 on oral glucose tolerance test (OGTT) and AUC in STZ/HFD-induced diabetic mice. (**A**) Blood glucose levels were measured at 0, 30, 60, and 120 min after oral glucose administration. (**B**) Glucose AUC values were calculated from the OGTT data. The STZ/HFD group showed significantly higher glucose levels and AUC values compared to the Normal group. BE-FD-1 (500 mg/kg) and Metformin treatment significantly reduced AUC compared to the STZ/HFD group. Data are presented as the mean ± SEM (n = 5 per group). Statistical analysis was performed using one-way ANOVA followed by Tukey’s post hoc test (# *p* < 0.0001 vs. Normal, *** *p* < 0.001 vs. STZ/HFD). The circles indicate individual data points, and the × symbols indicate the mean values. Some lines/data series overlap due to the presentation of multiple parameters in the same graph; this does not affect the interpretation of the results.

In the OGTT, the STZ/HFD group exhibited a marked elevation in blood glucose levels and delayed recovery compared with the normal group. In contrast, BE-FD-1 administration attenuated the glucose response, particularly at 500 mg/kg. AUC analysis revealed that the STZ/HFD group had an AUC value of 28,725, whereas the BE-FD-1 500 mg/kg group showed a reduced value of 23,667 (−17.6%) (Figure 4A). The metformin-treated group showed an AUC of 23,658. BE-FD-1-sc also significantly reduced the glucose AUC compared with the STZ/HFD group (*** *p* < 0.001), indicating that BE-FD-1-sc retained glucose tolerance-improving activity (Figure 4B). However, the effect of BE-FD-1-sc appeared less pronounced than that of BE-FD-1 500 mg/kg. Both BE-FD-1 500 mg/kg and metformin significantly reduced the glucose AUC compared to the STZ/HFD group (*** *p* < 0.001), while the STZ/HFD group showed significantly higher glucose levels and AUC values than the normal group (# *p* < 0.0001).

### 3.5. Effect of BE-FD-1 on Liver Function Markers

**Figure 5:**
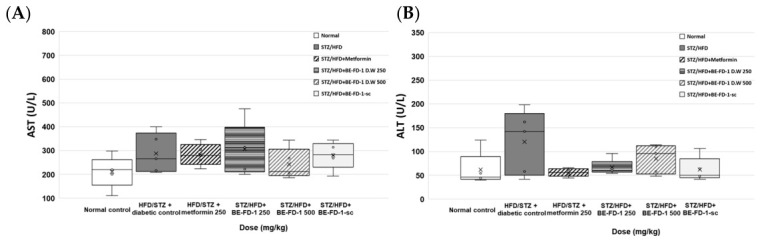
Effect of BE-FD-1 on serum AST levels in STZ/HFD-induced diabetic mice. Serum AST (**A**) and ALT (**B**) levels were measured at the end of the experimental period. Data are presented as the mean ± SEM (n = 5 per group). Statistical significance was analyzed using one-way ANOVA followed by Tukey’s post hoc test. The circles indicate individual data points, and the × symbols indicate the mean values. Some lines/data series overlap due to the presentation of multiple parameters in the same graph; this does not affect the interpretation of the results.

Serum AST and ALT levels were elevated in the STZ/HFD group compared with those in the normal control group. Serum AST levels were 199.6 U/L in the normal control group and 286.4 U/L in the STZ/HFD group, while the BE-FD-1 500 mg/kg group showed an AST level of 242.8 U/L, representing a 15.2% lower value than that of the STZ/HFD group (Figure 5A). Nevertheless, because this difference was not statistically significant, the AST-related finding should be interpreted cautiously. BE-FD-1-sc did not markedly alter serum AST or ALT levels compared with the STZ/HFD group, indicating that its effect on liver-related biochemical parameters was limited under the present experimental conditions. In contrast, ALT was significantly reduced in the BE-FD-1 500 mg/kg group compared with the STZ/HFD group (Figure 5B), indicating that the effect of BE-FD-1 on liver-related biochemical parameters was more evident for ALT than for AST.

### 3.6. Effect of BE-FD-1 on Serum Lipid Profiles

**Figure 6:**
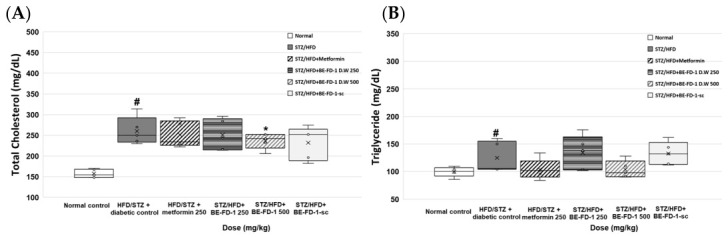
Effect of BE-FD-1 on serum lipid profile in STZ/HFD-induced diabetic mice. Serum total cholesterol (**A**) and triglyceride (**B**) levels were measured at the end of the experimental period. Data are presented as the mean ± SEM (n = 5 per group). Statistical significance was analyzed using one-way ANOVA followed by Tukey’s post hoc test (# *p* < 0.0001 vs. Normal, * *p* < 0.001 vs. STZ/HFD). The circles indicate individual data points, and the × symbols indicate the mean values. Some lines/data series overlap due to the presentation of multiple parameters in the same graph; this does not affect the interpretation of the results.

Serum triglyceride levels were 124.8 mg/dL in the STZ/HFD group, higher than 99.6 mg/dL in the normal group. The BE-FD-1 500 mg/kg group showed 103.6 mg/dL (−17.0% vs. STZ/HFD). The metformin-treated group exhibited 104.0 mg/dL, whereas the BE-FD-1 250 mg/kg group showed 134.0 mg/dL. BE-FD-1-sc did not markedly reduce serum triglyceride levels compared with the STZ/HFD group, suggesting that its triglyceride-lowering effect was limited under the present experimental conditions. Triglyceride levels were significantly higher in the STZ/HFD group than in the normal group and were significantly reduced by BE-FD-1 500 mg/kg and metformin treatment compared with the STZ/HFD group(Figure 6A).

The serum total cholesterol levels were 260.0 mg/dL in the STZ/HFD group. The BE-FD-1 500 mg/kg group showed 236.8 mg/dL (−8.9% vs. STZ/HFD), while the metformin-treated group exhibited 251.2 mg/dL. Relatively higher levels were observed in the BE-FD-1 250 mg/kg group. BE-FD-1-sc also showed no apparent improvement in serum total cholesterol levels compared with the STZ/HFD group. Total cholesterol levels were significantly higher in the STZ/HFD group than in the normal group and were significantly decreased by BE-FD-1 500 mg/kg and metformin treatment compared with the STZ/HFD group(Figure 6B).

### 3.7. Effect of BE-FD-1 on Fasting Blood Glucose Levels

**Figure 7:**
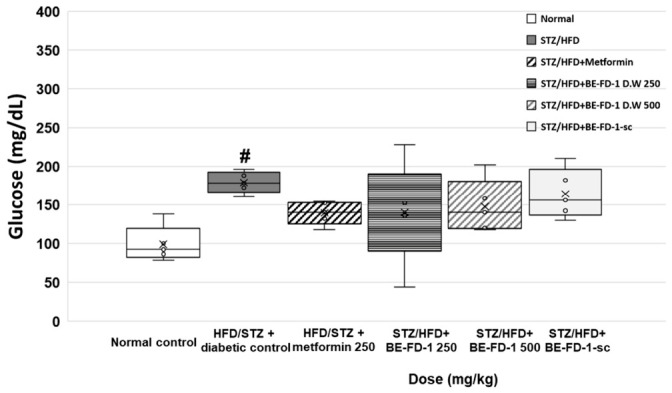
Effect of BE-FD-1 on fasting blood glucose levels in STZ/HFD-induced diabetic mice. Fasting blood glucose levels were measured at the end of the experimental period. Data are presented as the mean ± SEM (n = 5 per group). Statistical significance was analyzed using one-way ANOVA followed by Tukey’s post hoc test (# *p* < 0.0001 vs. Normal). The circles indicate individual data points, and the × symbols indicate the mean values. Some lines/data series overlap due to the presentation of multiple parameters in the same graph; this does not affect the interpretation of the results.

Fasting blood glucose analysis showed that levels in the STZ/HFD group were significantly elevated compared to those in the normal group. In contrast, fasting blood glucose levels in the BE-FD-1 500 mg/kg-treated group decreased by 31.2 mg/dL (−17.4%) compared with the STZ/HFD group. A reduction in fasting glucose levels was also observed in the metformin-treated group, and the BE-FD-1 500 mg/kg group showed glucose levels within a range similar to that of the metformin group. Compared with the BE-FD-1 250 mg/kg group, lower fasting glucose levels were observed in the BE-FD-1 500 mg/kg-treated group. BE-FD-1-sc showed only a limited reduction in fasting blood glucose compared with the STZ/HFD group, and its effect was less pronounced than that of BE-FD-1 500 mg/kg. Fasting blood glucose levels were significantly higher in the STZ/HFD group than in the normal group (Figure 7).

### 3.8. Effect of BE-FD-1 on Serum Creatinine Levels

**Figure 8:**
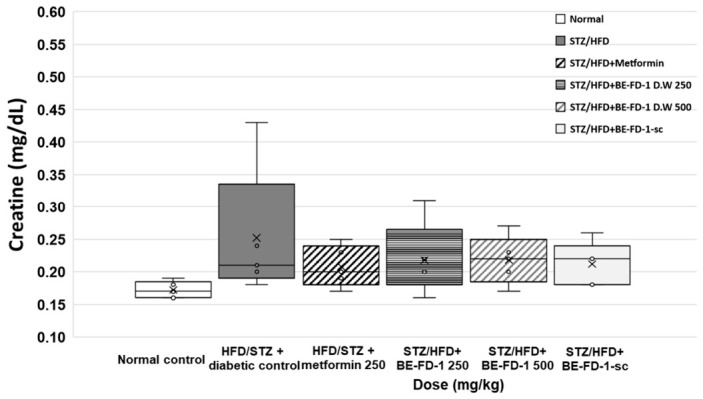
Effect of BE-FD-1 on serum creatinine levels in STZ/HFD-induced diabetic mice. Serum creatinine levels were measured at the end of the experimental period. Data are presented as the mean ± SEM (n = 5 per group). Statistical significance was analyzed using one-way ANOVA followed by Tukey’s post hoc test. The circles indicate individual data points, and the × symbols indicate the mean values. Some lines/data series overlap due to the presentation of multiple parameters in the same graph; this does not affect the interpretation of the results.

Creatinine analysis showed that the creatinine level in the STZ/HFD group was 0.25 mg/dL, which was increased compared with the normal group (0.17 mg/dL). In contrast, the BE-FD-1 500 mg/kg-treated group showed a creatinine level of 0.22 mg/dL, representing a decrease of 0.03 mg/dL (−12.0%) compared with the STZ/HFD group. The creatinine level in the BE-FD-1 500 mg/kg group was comparable to that of the metformin-treated group (0.21 mg/dL) and the BE-FD-1 250 mg/kg group (0.22 mg/dL). BE-FD-1-sc also did not markedly alter serum creatinine levels compared with the STZ/HFD group. Creatinine levels remained within the normal range in all treatment groups, and no statistically significant differences were observed among the treatment groups (Figure 8). However, because additional renal biomarkers and renal histopathological analyses were not performed, this finding should be interpreted only as the absence of an overt creatinine-based renal toxicity signal under the present experimental conditions.

### 3.9. Effect of BE-FD-1 on Serum Insulin Levels

**Figure 9:**
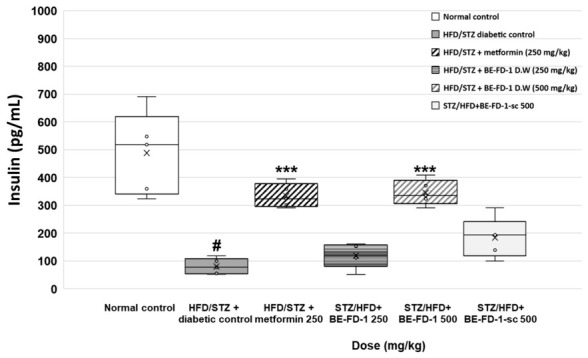
Effect of BE-FD-1 on serum insulin levels measured by ELISA in STZ/HFD-induced diabetic mice. Serum insulin levels were measured at the end of the experimental period using ELISA. Data are presented as the mean ± SEM (n = 5 per group). Statistical significance was analyzed using one-way ANOVA followed by Tukey’s post hoc test (# *p* < 0.0001 vs. Normal, *** *p* < 0.001 vs. STZ/HFD). The circles indicate individual data points, and the × symbols indicate the mean values. Some lines/data series overlap due to the presentation of multiple parameters in the same graph; this does not affect the interpretation of the results.

Serum insulin analysis showed that the insulin level in the STZ/HFD group was 80.2 pg/mL, which was significantly lower than that in the normal group (488.2 pg/mL). In contrast, the BE-FD-1 (500 mg/kg)-treated group exhibited an insulin level of 345.9 pg/mL, indicating a significant restoration compared with the STZ/HFD group. The insulin level in the BE-FD-1 (500 mg/kg) group was numerically comparable to that in the metformin-treated group (334.2 pg/mL), although no statistically significant difference was observed between the two groups. BE-FD-1-sc did not markedly restore serum insulin levels compared with the STZ/HFD group, suggesting that its insulin-related effect was limited under the present experimental conditions (Figure 9).

## 4. Discussion

T2DM is a multifactorial metabolic disorder characterized by insulin resistance, impaired β-cell function, chronic hyperglycemia, and associated dyslipidemia, all of which contribute to progressive systemic organ damage [1,2]. In the present study, the antidiabetic potential of BE-FD-1, a functional material derived from dried leaves of *R. sativus* L., was comprehensively evaluated in an HFD/STZ-induced T2DM mouse model. Administration of BE-FD-1, particularly 500 mg/kg, improved multiple diabetes-related metabolic parameters, including body weight gain, glucose tolerance, serum insulin, and selected biochemical markers. Collectively, these findings suggest that BE-FD-1 holds considerable promise and may have potential as a natural functional ingredient in the management of T2DM.

ICP-MS analysis confirmed the presence of vanadium, chromium, magnesium, zinc, and calcium in radish leaf samples collected during the cultivation process. The variability observed between the two QC phases may reflect differences in sampling time, cultivation stage, and agronomic conditions, all of which are known to influence mineral accumulation in biofortified crops [28]. These data were used primarily for cultivation-stage mineral verification rather than as a direct measure of in vivo mineral exposure following oral administration, because mineral concentration in plant tissue does not directly indicate bioaccessibility or bioavailability [29]. Among the identified minerals, zinc and magnesium are known to support insulin signaling and glucose uptake, whereas chromium and vanadium have been reported to exhibit insulin-mimetic or insulin-sensitizing properties [32,33]. Therefore, the antidiabetic effects observed in the present study may, at least in part, reflect the contribution of these minerals in addition to the intrinsic bioactive constituents of radish leaves.

It should also be noted that some of the absolute biomarker values observed in the present study may appear higher than those reported in other HFD/STZ-based studies. Such differences may arise from variations in diet composition, duration of HFD exposure, STZ dosing regimen, age and body weight of the animals, fasting duration before sampling, timing of blood collection, and analytical assay platform [34,35]. Therefore, the endpoint values in the present study are most appropriately interpreted in terms of within-study group comparisons rather than direct numerical equivalence across all published HFD/STZ models.

With regard to body weight, the STZ/HFD-induced diabetic group showed a tendency toward greater body weight gain than the normal control group during the experimental period. BE-FD-1 treatment, particularly at 500 mg/kg, tended to attenuate body weight gain. However, this finding should be interpreted cautiously because clear statistical significance was not indicated. Therefore, the body weight-related findings should be regarded as numerical tendencies only and should not be interpreted as definitive evidence of body weight-regulating activity. Previous HFD/STZ-based studies have reported variable body weight responses depending on the experimental design and intervention [7,36]. Because food intake, water intake, and physical activity were not monitored in the present study, the body weight-related findings should be interpreted with caution. Moreover, plant-derived polyphenol-rich preparations and mineral-related interventions have been reported to influence energy balance-related parameters, including food intake, water consumption, locomotor activity, energy expenditure, fatty acid oxidation, or satiety-related signals, depending on the experimental model and intervention [37,38,39,40]. Therefore, future studies should include systematic monitoring of food intake, water intake, spontaneous activity, and energy expenditure to determine whether the metabolic effects of BE-FD-1 are independent of altered energy intake or expenditure.

With respect to glucose metabolism, the OGTT results demonstrated that BE-FD-1 at 500 mg/kg significantly reduced the glucose area under the curve (AUC) by 17.6% compared to the STZ/HFD group, achieving a level of glycemic improvement comparable to that of the metformin-treated group. This improvement may be partly associated with delayed carbohydrate digestion and absorption, as supported by the α-glucosidase-inhibitory activity observed in the present study and by previous reports on Raphanus sativus-derived materials [15]. In addition, previously reported activities of plant-derived polyphenols and flavonoids suggest their potential relevance to postprandial glycemic control; however, insulin signaling-related proteins or molecular markers were not evaluated in the present study. Therefore, possible modulation of insulin signaling should be regarded as a hypothesis requiring further validation rather than a mechanism directly demonstrated in this study. In parallel, fasting blood glucose levels were reduced by 17.4% in the BE-FD-1 500 mg/kg group compared to those in the STZ/HFD group, further supporting the glucose-lowering potential of BE-FD-1.

Nevertheless, because insulin concentrations were not measured during OGTT, the present study could not distinguish whether the improvement in glucose tolerance was primarily related to enhanced insulin secretion, improved insulin sensitivity, delayed intestinal glucose absorption, or a combination of these mechanisms.

Among the notable findings of the present study was the significant restoration of serum insulin levels observed in the BE-FD-1 500 mg/kg group (345.9 pg/mL), which was markedly higher than that of the STZ/HFD group (80.2 pg/mL) and comparable to the level observed in the metformin-treated group (334.2 pg/mL). STZ is well known to impair pancreatic β-cell function through DNA alkylation [8], resulting in reduced insulin secretory capacity. Therefore, the increased serum insulin level observed in BE-FD-1-treated mice may suggest an improvement in circulating insulin availability under the present experimental conditions. Previous studies have reported that polyphenols and glucosinolate-related compounds from plant-derived materials are associated with oxidative stress modulation and insulin-related metabolic regulation [41,42]. However, their relevance to the present findings remains hypothetical. Because pancreatic histology, β-cell mass assessment, insulin immunohistochemistry, and insulin signaling analyses were not performed, direct preservation, protection, regeneration, or functional recovery of pancreatic β-cells cannot be concluded from the current data alone.

Regarding hepatic function, the STZ/HFD group exhibited significantly elevated serum ALT and AST levels, reflecting hepatocellular damage induced by chronic hyperglycemia and ectopic lipid accumulation [43]. BE-FD-1 (500 mg/kg) significantly reduced serum ALT levels, whereas AST levels showed a non-significant decreasing trend. The differential response between ALT and AST may be explained by the distinct subcellular origins of these two enzymes: ALT is predominantly cytosolic in distribution and more specific to hepatocellular injury, whereas AST is broadly distributed across multiple tissues, including cardiac and skeletal muscles [44]. The ALT-related biochemical improvement of BE-FD-1 may be mediated by the antioxidant capacity of its polyphenolic constituents, which attenuates reactive oxygen species (ROS)-mediated hepatocyte damage. Previous studies have demonstrated that *R. sativus* extracts reduce serum ALT and AST levels in liver injury models by suppressing oxidative stress and inhibiting inflammatory cytokine production [45]. Because AST did not show a statistically significant reduction, the liver-related findings should also be interpreted conservatively.

With respect to lipid metabolism, BE-FD-1 at 500 mg/kg significantly reduced serum triglyceride and total cholesterol levels by 17.0% and 8.9%, respectively, compared to the STZ/HFD group. HFD/STZ-induced diabetic mice develop dyslipidemia, characterized by elevated triglyceride and cholesterol levels, arising from impaired lipid metabolism and reduced insulin-mediated lipoprotein lipase activity [5]. The lipid-lowering effects observed in the BE-FD-1 500 mg/kg group may be partly explained by previously reported activities of *R. sativus*-derived materials, including effects on lipid metabolism, cholesterol excretion, and adipokine-related regulation [19,46]. However, hepatic lipogenesis, fatty acid β-oxidation, adiponectin levels, and related molecular markers were not evaluated in the present study. Therefore, the involvement of these pathways remains hypothetical and requires further validation. These results are consistent with those of previous reports, demonstrating that *R. sativus* var. caudatus extract significantly reduced hepatic total cholesterol and triglyceride concentrations in HFD-induced obese mice [20].

Serum creatinine levels in all treatment groups remained within the normal range throughout the experimental period, and no statistically significant differences were observed between groups. Although the STZ/HFD group exhibited a slightly elevated creatinine level (0.25 mg/dL) relative to the normal group (0.17 mg/dL), the BE-FD-1 500 mg/kg group maintained a creatinine level (0.22 mg/dL) comparable to those of both the metformin-treated group (0.21 mg/dL) and the BE-FD-1 250 mg/kg group (0.22 mg/dL). These findings indicate that no overt renal toxicity was observed based on serum creatinine under the present experimental conditions, thereby supporting its preliminary safety profile [47]. Nevertheless, a more comprehensive safety evaluation, including additional renal biomarkers and histological analyses, will be required in future studies.

Taken together, the results of the present study demonstrate that BE-FD-1-derived *R. sativus* L. dried leaves exert multi-targeted antidiabetic effects in HFD/STZ-induced T2DM mice, including the amelioration of body weight gain, glucose intolerance, insulin deficiency, hepatic injury, and dyslipidemia, without apparent nephrotoxicity. These beneficial effects may be associated with the previously reported biological activities of *R. sativus*-derived constituents, including dietary fiber, polyphenols, glucosinolates, and β-carotene; however, the specific active constituents and molecular mechanisms responsible for the observed effects were not directly identified in the present study [14,15]. The dose-dependent effects of BE-FD-1 at 500 mg/kg consistently demonstrated superior efficacy relative to the 250 mg/kg dose across all evaluated parameters, further corroborating the biological activity of BE-FD-1.

Several limitations should be considered when interpreting the present findings. First, this study was designed as an initial exploratory in vivo efficacy study to determine whether BE-FD-1 produces detectable antidiabetic signals in the HFD/STZ-induced diabetic mouse model. Therefore, the relatively small sample size (n = 5 per group) may have reduced the statistical power for some endpoints and contributed to non-significant findings despite numerical trends. Although BE-FD-1 at 500 mg/kg significantly improved several metabolic endpoints, including glucose AUC, fasting blood glucose, serum insulin, ALT, triglyceride, and total cholesterol, the overall effects should be interpreted as preliminary and moderate rather than definitive or strong. In particular, body weight gain and AST showed only non-significant tendencies and should not be overinterpreted.

Second, although additional compositional analyses were performed in the revised study, the final BE-FD-1 test material remains incompletely standardized. Additional ICP-MS analysis was conducted to provide mineral composition data for BE-FD-1-related samples, and total polyphenol and total flavonoid contents were evaluated as additional plant-derived compositional parameters.however, they do not replace comprehensive extract standardization. Detailed extraction parameters, including extraction temperature, extraction time, extraction yield, long-term stability, and batch-to-batch reproducibility, were not fully established. In addition, comprehensive phytochemical profiling of the final BE-FD-1 extract using HPLC or LC-MS was not performed, and major phytochemical constituents, including glucosinolates, flavonoids, polyphenols, and β-carotene, were not individually quantified. Therefore, mechanistic interpretations based on these compounds should be considered literature-based hypotheses rather than direct evidence from the present study.

Third, the present study did not include a true non-fortified *Raphanus sativus* extract-treated animal control group, which remains a major limitation of the experimental design. To partially address this issue, we incorporated the animal efficacy results of a non-fortified *Raphanus sativus* leaf sample obtained through subsequent cultivation without additional mineral treatment after the initial BE-FD-1 mineral-fortification cultivation. This sample showed lower levels of several key minerals, including V, Cr, Mg, and Zn, compared with BE-FD-1, whereas Ca remained comparable. In parallel, it exhibited relatively weaker or less consistent metabolic effects than BE-FD-1 500 mg/kg in HFD/STZ-induced diabetic mice. Although these findings provide supportive information suggesting a possible association between cultivation-dependent mineral profile changes and biological activity, this comparison does not fully replace a true non-fortified extract-treated animal control group. Therefore, the relative contributions of the radish leaf extract itself, the mineral-fortification process, and their combined action remain to be clarified in future studies.

Fourth, food intake, water consumption, and physical activity were not monitored during the experimental period. Therefore, the potential contribution of altered caloric intake, water intake, or energy expenditure to the observed changes in body weight gain, glucose metabolism, and downstream biochemical parameters cannot be fully excluded. Future studies should include food and water intake monitoring, spontaneous activity assessment, energy expenditure analysis, and, where appropriate, pair-feeding designs.

Fifth, the mechanisms underlying the metabolic effects of BE-FD-1 were not directly investigated. Insulin was measured only as an endpoint serum parameter, and OGTT-coupled insulin measurements, insulin tolerance testing, HOMA-IR analysis, pancreatic histology, β-cell mass analysis, insulin immunostaining, insulin signaling markers, and intestinal glucose absorption-related assays were not performed. Therefore, the improved glucose tolerance observed in BE-FD-1-treated mice cannot be attributed specifically to enhanced insulin secretion, improved insulin sensitivity, delayed intestinal glucose absorption, or β-cell preservation. These mechanistic explanations should be regarded as hypothetical and require further validation.

Sixth, mineral bioavailability, bioaccessibility, systemic exposure, and tissue distribution were not assessed. Therefore, although the mineral profile of BE-FD-1 suggests the presence of fortified minerals such as V, Cr, Mg, Zn, and Ca, the direct contribution of individual minerals to the observed metabolic effects cannot be determined under the current experimental conditions. Future studies should include mineral bioaccessibility assays, plasma and tissue mineral analysis, and pharmacokinetic or tissue distribution assessments.

Seventh, the safety evaluation was limited. Although serum creatinine levels did not indicate an overt creatinine-based renal toxicity signal under the present experimental conditions, broader renal biomarkers, urinary parameters, organ weight analysis, hematology, and renal or hepatic histopathology were not evaluated. Therefore, no definitive safety conclusion can be drawn from the present study. Future studies should include comprehensive toxicological assessments, including BUN, urinary albumin, kidney injury markers, organ weights, histopathology, and longer-term repeated-dose safety evaluation.

Finally, this study was restricted to male C57BL/6 mice and used a relatively short HFD/STZ induction protocol to generate a T2DM-like metabolic phenotype. Therefore, potential sex-based differences, long-term metabolic responses, and broader translational relevance remain to be investigated. Collectively, the present findings should be interpreted as exploratory evidence that BE-FD-1 may exert moderate metabolic benefits in HFD/STZ-induced diabetic mice. Further confirmatory studies using larger cohorts, formal sample size estimation, standardized BE-FD-1 preparations, true non-fortified extract-treated controls, comprehensive compositional profiling, mechanistic validation, and broader safety assessment are required before the functional value of BE-FD-1 can be more firmly established.

In conclusion, BE-FD-1 derived from *R. sativus* L. demonstrated significant antidiabetic efficacy in an HFD/STZ-induced T2DM mouse model, with effects across multiple metabolic parameters that were directionally similar to those observed with metformin, particularly at the 500 mg/kg dose. These findings provide a scientific basis for the development of BE-FD-1 as a natural functional ingredient candidate for further investigation in the context of type 2 diabetes-related metabolic dysfunction. However, additional studies with larger sample sizes, more comprehensive metabolic phenotyping, and mechanistic analyses are required.

## 5. Conclusions

In conclusion, BE-FD-1 showed preliminary metabolic improvement signals in an HFD/STZ-induced diabetic mouse model, particularly at 500 mg/kg, as indicated by improvements in glucose tolerance, fasting blood glucose, serum insulin, and selected lipid-related parameters. However, these findings should be interpreted with caution because of the exploratory study design, small sample size, incomplete standardization and characterization of the final extract, the absence of a true non-fortified extract-treated animal control group, lack of food intake, water intake, and physical activity monitoring, limited mechanistic validation, and limited safety assessment. Therefore, the present findings do not establish the functional value of BE-FD-1 at this stage, but rather provide early experimental evidence supporting further investigation. Future confirmatory studies using larger cohorts, standardized BE-FD-1 preparations, comprehensive phytochemical and mineral profiling, mechanistic analyses, and broader toxicological evaluation are required before the functional applicability of BE-FD-1 can be more confidently considered.

## Figures and Tables

**Table 1:** Mineral composition of radish leaf samples analyzed during the cultivation-stage QC process by ICP-MS.

QC Phase	Commission Date	Report No.	V (mg/kg)	Cr (mg/kg)	Mg (mg/kg)	Zn (mg/kg)	Ca (mg/kg)
QC-1	2 October 2024	2024-3345	14.1	2.17	2857	88.6	5640
QC-2	28 October 2024	2024-3684	3.36	0.28	4304	32.1	22,790
Control	5 May 2026	2026-1590, 1591	0	0.22	1899	13.2	7551
BE-FD-1	10 October 2024; 5 May 2026	2024-3345, 3684, 2026-1594	5.9	0.88	2804	48.2	12,396
BE-FD-1-sc	5 May 2026	2026-1592, 1593	0.12	0.51	1223	12.2	13,040

**Table 2:** Chemical profile of BE-FD-1 extract by UPLC-QTOF-HRMS.

Tentative Compounds	Retention Time (min)	Formula	Theoretical (*m*/*z*)	Experimental (*m*/*z*)	Deprotonated Ones or Adducts	Δ (ppm) ^a^
Quercetin 3,7-di-O-rhamnoside	5.710	C_27_H_30_O_15_	595.1657	595.1663	[M + H]^+^	1.01
Kaempferitrin	6.290	C_27_H_30_O_14_	579.1708	579.1714	[M + H]^+^	1.04
Kaempferol-7-O-rhamnoside	6.290	C_21_H_20_O_10_	433.1129	433.1135	[M + H]^+^	1.39

^a^ Deviation of measured *m*/*z* from calculated *m*/*z* values for a pseudomolecular ion generated from the molecular formula.

## Data Availability

All data generated or analyzed during this study are included in this published article. Further information may be obtained from the corresponding author upon reasonable request.
